# The danger of the toxicity and inefficacy of alcohol-based hand rubs in Iran during COVID-19: a cross-sectional study

**DOI:** 10.1186/s13756-023-01244-w

**Published:** 2023-04-25

**Authors:** Ali Rafizadeh, Ali-Asghar Kolahi, Shahab Shariati, Nasim Zamani, Darren M Roberts, Hossein Hassanian-Moghaddam

**Affiliations:** 1 Faculty of Nursing & Midwifery, Rasht Branch, Islamic Azad University, Rasht, Iran; 2 Social Determinant of Health Research Center, Shahid Beheshti University of Medical Sciences, Tehran, Iran; 3 Department of Chemistry, Rasht Branch, Islamic Azad University, Rasht, Iran; 4 Department of Internal Medicine, St. Agnes Medical Center, Fresno, CA USA; 5 Edith Collins Centre, Drug Health Services, Royal Prince Alfred Hospital, Camperdown, NSW Australia; 6 Department of Clinical Toxicology, School of Medicine, Shohada-e-Tajrish Hospital, Shahid Beheshti University of Medical Sciences, Tehran, Iran

**Keywords:** COVID-19, Hand sanitizer, Hand rubbers, Methanol, Methanol intoxication, Methanol detection, Methanol determination

## Abstract

**Background:**

The use of disinfectants and alcohol-based hand rubs (ABHR) to prevent COVID-19 transmission increased in the first wave of the infection. To meet the increased demand, the Iranian Ministry of Health issued an emergency use authorization allowing new manufacturers to enter the market, despite the limited capacity for surveillance of these products during COVID-19. Methanol poisoning outbreaks spread rapidly, and more people died from methanol poisoning than COVID-19 in some cities. The aim of this study was to analyze some ABHRs in the Iranian market to see if (a) ABHRs are standard and suitable for hand antisepsis and (b) contained potentially dangerous toxic alcohols.

**Method:**

Between February and March 2020, 64 brands of ABHR were conveniently collected from pharmacies, supermarkets, and shops selling hygienic products and analyzed using Gas Chromatography. World Health Organization and Food and Drug Administration guidelines were used to define minimum requirements for ABHR. For estimating the risk for acute methanol poisoning, we assumed a serum methanol concentration of 200 mg/L following ABHR ingestion was sufficient to cause intoxication. This threshold concentration would be achieved in an average 75-kg adult after consuming 8000 mg (or eight grams) methanol in 1–2 h.

**Results:**

The median [IQR] (range) concentration of ethanol, isopropanol, and methanol were 59% v/v [32.2, 68] (0, 99), 0 mg/L [0, 0] (0, 197,961), and 0 mg/L [0, 0] (0, 680,100), respectively. There was a strong negative correlation between methanol and ethanol contents of hand rubbers (r= -0.617, p < 0.001). Almost 47% of ABHRs complied with minimum standards. In 12.5% of ABHRs, high concentrations of methanol were observed, which have no antiseptic properties but could cause acute methanol poisoning if ingested.

**Conclusion:**

COVID-19 initiated a policy for distribution and use of ABHR with little control. As ABHR and masks are still accepted preventive measures of the disease, non-standard ABHR compositions may increase the population’s risk to both COVID-19 infection and methanol poisoning.

## Introduction

On March 11th, 2020, the COVID-19 pandemic was declared by the World Health Organization (WHO) [1, 2]. Since there was no specific treatment at that time, health authorities recommended frequent use of alcohol-based hand rubs (ABHR) to prevent the spread of the disease [3–6].

ABHR is a liquid, gel or foam with antiseptic properties used to reduce microorganisms on the hands and prevent their transmission [7, 8]. ABHR may be preferred over washing hands with soap and water due to ease of use, improved tolerance, antiseptic effects targeting microorganisms, and other factors [8–10]. Except for spores, ABHRs have suitable effects against all microorganisms (such as ‘flu, common cold, corona virus, and HIV) [2, 3, 11]. ABHRs are a combination of different alcohols such as ethanol and isopropyl alcohol with alcohol concentrations of at least 60 to 95%. Some sanitizers contain compounds such as glycerol to prevent drying of the skin [12, 13]. Methanol is banned from use in ABHR because it is highly toxic if consumed and is ineffective at destroying microorganisms [14].

WHO suggested two formulations, considering cost and microbicidal activity.

The only difference is the main alcohol, where 1000 ml contains either 833.3 ml of ethanol 96% v/v, or 751.5 ml of isopropyl alcohol 99.8%. The remaining ingredients in both formulations are the same; 41.7 ml of hydrogen peroxide (H_2_O_2_) 3%, 14.5 ml of glycerol 98%, and distilled water to make 1000 ml ABHR [15].

Drinking alcohol (ethanol) is banned in Iran and therefore, due to illegal importations outside of regulatory processes, the more toxic alcohol methanol is often mis-sold as alcohol and consumed. Unfortunately, therefore, methanol intoxication is a common health problem in Iran. In previous years, alcohol-producing factories had to add 5% v/v (50,000 mg/L) methanol to the products to make them non-potable. However, this policy was later changed to the addition of a highly bitter substance named “Bitrex” (*Chemical name: Denatonium benzoate*) due to the extensive poisoning caused by the ingestion of methanol. However, this problem has never been completely eradicated, and the risk of methanol poisoning remains [16–19].

The booming market for ABHR during the outbreak of COVID-19 caused an increase in the production of non-standard alcohols to enter the consumer market. In addition, the de-colorization of industrial alcohol, which is another type of denatured alcohol, with sodium hypochlorite to gain more profits exacerbated the situation. These industrial alcohols may have high concentrations of methanol (even more than 90% v/v or 900,000 mg/L) and some warning chemical additives including coloring, odor and bittering substances like pyridine, turpentine, and denatonium benzoate, respectively [20]. At the same time, worldwide social rumors that drinking alcohol is protective against COVID-19 led to an increase in acute methanol poisoning during COVID-19 in Iran [21–24], many cases of which were reported in children and adolescents and more people died from methanol poisoning than COVID-19 in some cities [21–23].

The aim of this study was to analyze products sold as ABHR in the Iranian market to describe the prevalence of products containing (a) standard alcohols suitable for antiseptic use, and (b) those containing potentially dangerous toxic alcohols.

## Materials and methods

Between February and March 2020, 64 different brands of ABHR were conveniently collected from pharmacies, supermarkets, and hygienic products shops. These ABHR samples were both registered and unregistered brands from different local commercial stores (Rasht, Gilan province, and Tehran, Iran). Some of these products had commercial labels with information on the chemical ingredients including the concentrations of alcohols.

Samples were analyzed using Gas Chromatography (GC) to detect and measure ethanol, isopropanol and methanol [25, 26]. We classified a product as an alcohol if the concentration was more than the limit of GC quantification (1 mg/L).

### Apparatus

A GC device (YL 6100 GC model, South Korea) was used to determine methanol, ethanol, and isopropyl alcohol concentrations. The GC system was equipped with a flame ionization detector (FID) and TR-CN100, capillary column (60 m×0.25 mm×0.2 μm). A 10-µL Hamilton syringe was used to inject samples [18].

Helium carrier gas with a flow rate of 2 ml/min was used for alcohols separation. Two micro liters of all standards and samples were injected (with 1:40 split ratio) to GC apparatus as triplicate at column without pre-incubator temperature in isothermal condition. The injector, oven and FID detector temperatures were fixed at 220, 80, and 230 degrees Celsius, respectively. The obtained results were corrected based on internal standard peak and finally, the average of three replicate results were used as last results for next calculations [27].

### Chemicals

The required methanol, ethanol, isopropyl alcohol, and 1-butanol for preparation of standard solutions for GC method was prepared with analytical grade from Merck (Darmstadt, Germany) and used without further purification. De-ionized double distillated water (D.W) was used for preparation of all standard solutions and dilution of the ABHR samples.

### Preparation

Five mixed standards with 0–6,400 mg/L concentrations of methanol, ethanol, and isopropyl alcohol were prepared by a serial method to evaluate their contents. Also, three solutions were prepared with 2,500, 5,000, and 10,000 mg/L of ethanol, methanol, and isopropyl alcohol in D.W to control results. All ABHR samples were also diluted by D.W (with a 1:100 ratio) to perform tests. Aqueous 1-butanol solution was added to all test tubes containing standards, control solutions, and samples as internal standard to attain 100 mg/L concentration.

### Standard alcohol-based hand rubs

World Health Organization and Food and Drug Administration (FDA) temporary guidelines were used to define minimum requirements for ABHR as the Gold Standard [5, 13]. The ABHR product was considered acceptable if it contained either ethanol or isopropanol at a minimum 60 or 70% v/v concentration, respectively [13].

### Potential for acute methanol poisoning by ABHR

We ignored any methanol level less than 630 mg/L, according to FDA temporary policy issued during the COVID-19 pandemic [13].

We assumed a serum methanol concentration of 200 mg/L (20 mg/dL) as having a potential risk of severe poisoning if untreated [14]. To obtain this methanol concentration in an average 75-kg adult with about 40 L of body water would be possible after consuming 8,000 mg (8 g) methanol in 1–2 h. This is approximately equal to 10 mL of absolute methanol in 100 mL water (10% volume/volume or v/v). Therefore, the methanol/ethanol ratio in an ABHR should be more than 1:100 to be considered potentially toxic, if methanol ingestion is more than 8000 mg in a 75 Kg individual. This ratio is in full compliance with the European Union standard regarding the permitted amount of methanol in alcoholic beverages and it is equivalent to 10 g of methanol in 1000 g of absolute ethanol (or 4,000 mg in one liter of spirit with 40% v/v alcoholic strength) [16, 18, 19].

### Statistical analysis

Statistical Package for Social Sciences (SPSS) version 24 (IBM Corporations, Chicago, Ill, USA) was used for statistical analysis. Simple descriptive analysis was done using median [IQR] and range or frequency (%). A Person bivariate correlation analysis was done to see possible correlation between measured alcohols. A *P* value less than 0.05 was considered to be statistically significant.

## Results

All of the 64 ABHRs analyzed contained an alcohol, including 28 (44%) with ethanol only, and 2 (3%) with methanol only. There was no case of pure isopropanol. 34 (53%) ABHRs contained more than one alcohol, including 20 (31%) with both ethanol and methanol, 6 (9%) with both ethanol and isopropanol, and the final 8 (13%) products with a combination of ethanol, methanol and isopropanol.

### Ethanol and isopropanol concentration

**Fig. 1:**
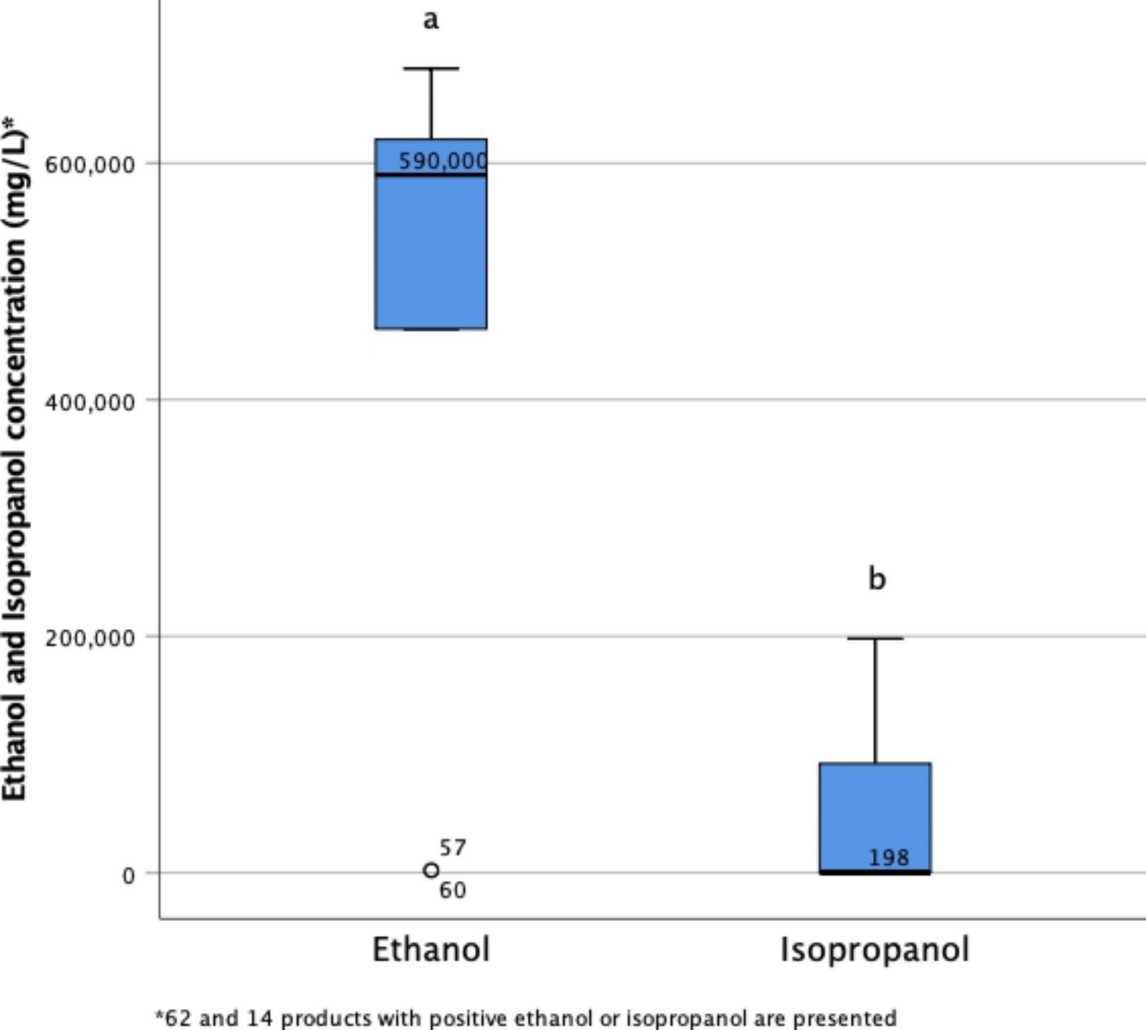
a) Ethanol concentration of alcohol-based hand sanitizers in 62 out of 64 samples collected during the first two-months of COVID-19 outbreak b) Isopropanol concentration of alcohol-based hand rubbers in the 14 out of 64 samples that contained isopropanol during the first two-months of COVID-19 outbreak

The median [IQR] (range) of ethanol concentration was 59% v/v (590,000 mg/L) [32.2, 68 v/v] (0, 99). The average ethanol concentration was lower than the minimum standard, Fig. 1a shows the boxplot distribution of the 62 products with positive ethanol (96.8% of those sampled). Only 30 out of 64 products (46.9% of those sampled) contained the minimum ethanol concentration of 60% v/v to be an effective ABHR and 10 (15.6%) had an ethanol concentration less than 0.5% v/v.

The median [IQR] (range) of isopropanol concentration was 0 mg/L [0, 0] (0, 197,961 mg/L). Figure 1b shows the boxplot distribution of the 14 products with positive isopropanol (21.9% of those sampled). Considering the maximum concentration of isopropanol in these ABHR samples, none of them were an effective ABHR.

### Methanol concentration

**Fig. 2:**
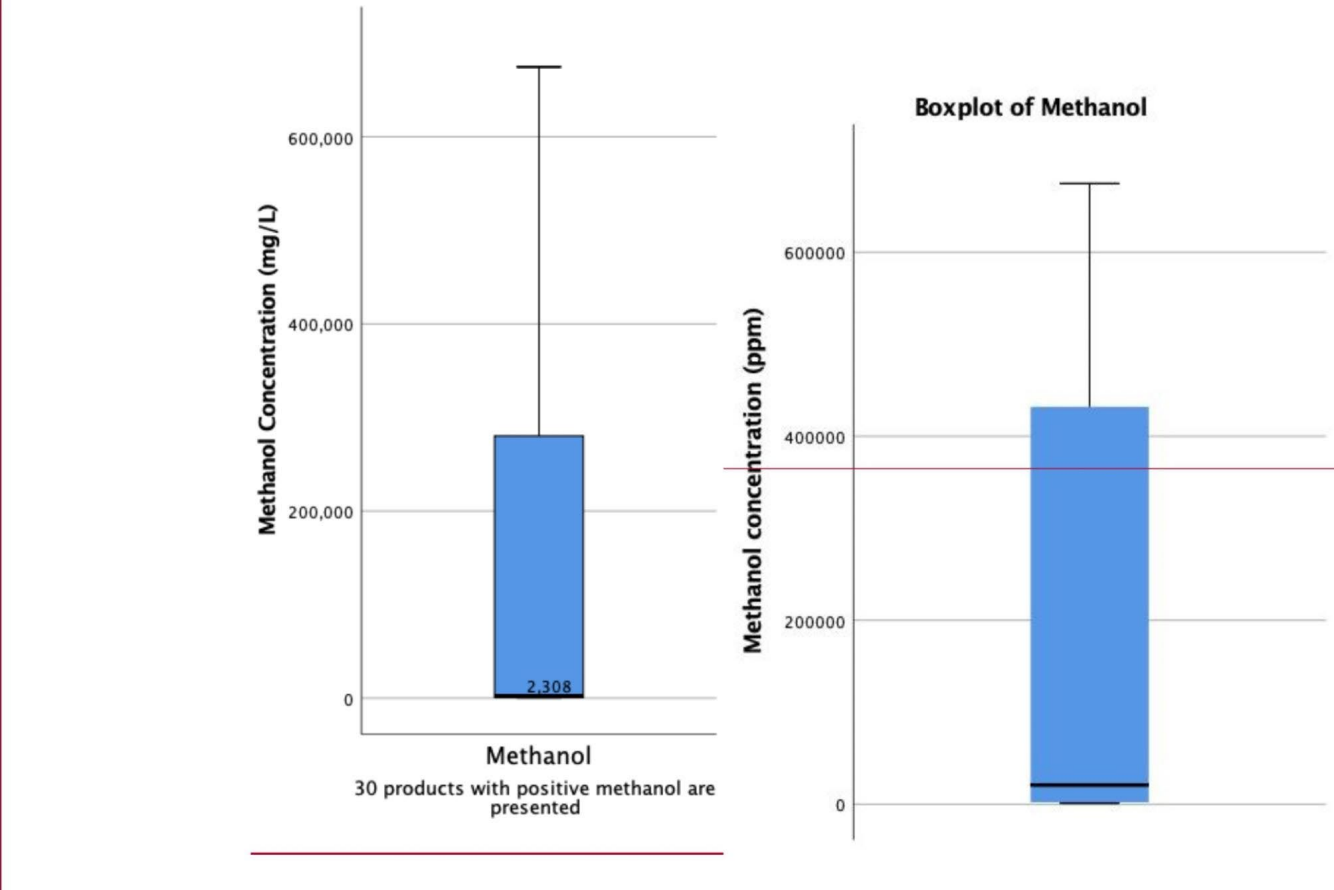
Methanol concentration of alcohol-based hand sanitizers in the 30 out of 64 samples that contained methanol during the first two-months of COVID-19 outbreak

The median [IQR] (range) of methanol concentration was 0 mg/L (0 ppm) [0, 0] (0, 680,100 mg/L). Thirty-four (53.1%) ABHR samples had methanol concentration < 50 mg/L and 22 (34.4%) had concentrations more than 630 mg/L (ppm). Figure 2 shows the boxplot distribution of methanol concentration in 22 products (34.4% of those sampled) with methanol concentration more than 630 mg/L. Eight (12.5%) of these samples were potentially toxic (more than 8,000 mg methanol in the absence of enough ethanol), being capable of causing high methanol serum concentrations and human toxicity if ingested (methanol/ethanol concentration must be > 1%,).

### Potential for acute methanol toxicity

There was a strong negative correlation between methanol and ethanol contents of ABHRs (r= -0.617, p < 0.001). According to the 1:100 ratio already described, eight (12.5%) of products had the potential to cause acute methanol toxicity if ingested. The methanol concentration in these samples ranged from 280,150 to 680,100 mg/L with an ethanol concentration of 0.1 to 26% (1,000 to 260,000 mg/L or 0.1–26% v/v). Table 1 shows the predicted serum methanol concentrations in these eight products if they were ingested over a short time.

**Table 1:** Risk of severe acute methanol toxicity in eight at-risk alcohol-based hand rubs (ABHRs) relative to the ingested volumes

Ethanol concentration of ABHR (v/v%)*¶	Methanol concentration of ABHR (mg/L)†	Methanol serum concentration (mg/L)^$^ if ingested (If weight = 75 Kg/ 40 L body water) between 1–2 h of a definitive volume					
		100 mL	200 mL	400 mL	600 mL	800 mL	1000 mL
22	280,150	700.38	1400.75	2801.50	4202.25	5603.00	7003.75
26	284,264	710.66	1421.32	2842.64	4263.96	5685.28	7106.60
1.8	343,292	858.23	1716.46	3432.92	5149.38	6865.84	8582.30
2.2	433,728	1084.32	2168.64	4337.28	6505.92	8674.56	10843.20
1	443,260	1108.15	2216.30	4432.60	6648.90	8865.20	11081.50
1	543,276	1358.19	2716.38	5432.76	8149.14	10865.52	13581.90
2.1	669,126	1672.82	3345.63	6691.26	10036.89	13382.52	16728.15
1.9	680,100	1700.25	3400.50	6801.00	10201.50	13602.00	17002.50

*Each 1% v/v is equal to 10,000 mg/L

¶The minimum effective concentration of ethanol is 60% v/v

†The maximum permitted concentration of methanol is 630 mg/L (FDA temporary policy) OK

^$^ the commonly quoted toxic threshold is serum concentration 200 mg/L

Table 2 summarizes the composition, inefficacy and potential toxicity of the 64 ABHRs products collected during COVID-19 in Tehran.

**Table 2:** Composition, inefficacy and potential toxicity of 64 alcohol-based hand rub (ABHR) products during COVID-19 in Tehran

	Alcohol component n (%)	Ineffective* (%)	Potentially toxic† n (%)
Ethanol	62 (97)	53	0
Isopropanol	14 (22)	100	0
Methanol	30 (47)	100	8 (12.5)
Any alcohol	64 (100)	53	8 (12.5)

*Inefficacy defined as ethanol < 60% or isopropanol < 70% †Toxicity defined as any methanol/ethanol concentration > 1%, while methanol ingestion is more than 8000 mg in a 75 Kg individual

## Discussion

The study shows that a high proportion of ABHRs were ineffective, or potentially toxic due to the methanol content. This was a result of uncontrolled sale of ABHR products in the absence of regulation, and potentially a lack of awareness by product manufacturers and/or distributors. We noted a strong negative correlation between ethanol and methanol concentrations, further increasing the risks of the methanol-based solutions for the reasons mentioned. .

An ABHR may contain one or more types of alcohol, with or without other excipients and humectants, to be applied on the hands to kill or suppress growth of microorganisms [12]. Our research shows only 50% of the studied ABHRs contain sufficient alcohol for antimicrobial properties. The Centers for Disease Control and Prevention (CDC) recommends using ABHR products that contain at least 60% ethyl alcohol (ethanol) or 70% isopropyl alcohol (isopropanol) in community settings [9]. In health care settings, CDC recommendations specify that these products should contain 60–95% alcohol (≥ 60% ethanol or ≥ 70% isopropanol) [4–6, 28, 29].

Due to the high concentration of alcohol (usually more than 40%) in ABHRs, higher than is normally found in alcoholic beverages, drinking ABHRs can lead to alcohol intoxication [5]. Several reports describe ingestion of such products in place of potable alcohol by patients with a history of mental illness or substance use disorders [3, 4]. There are reports of ABHRs being consumed for the purpose of intoxication, in particular in prisoners or hospitalized patients without access to potable alcohol [3].

According to the FDA, which regulates ABHR as an over-the-counter drug, methanol (methyl alcohol) is not an acceptable ingredient [6]. With the CDC recommending ABHR for preventative measures, many suppliers have increased production, or even shifted manufacturing lines to produce ABHR, during the COVID-19 pandemic [5].

Many countries including Iran have relaxed legislation to make it easier for local businesses to rapidly produce ABHR. The high demand for ABHR carried some potential risks including methanol poisoning, as we observed during the current COVID-19 outbreak [22–24].

The major route of exposure to people developing toxicity from ABHR is ingestion [2]. As shown in Table 1, ingestion of volumes as low as 100 mL can cause a serum methanol concentration as high as 1,700 mg/L from some products, almost 8.5 times the minimum 200 mg/L level of toxicity. The distribution of a huge amount of methanol-based ABHR in the community could contribute to the constant and numerous cases of methanol poisoning in Iran. Here, the number of cases poisoned by methanol has roughly doubled during the COVID-19 pandemic [21]. Time series analyses have shown that the numbers of methanol poisoning cases in Iran have not returned to the caseload pre- COVID-19 pandemic, and we are still facing methanol poisoning outbreaks in different cities [23]. This, as previously reported, may be contributed to by the social and mental stressors the people are experiencing. However, it cannot be denied that the poor control of the Iranian black market for alcoholic beverages, as well as the lack of quality control over production of ABHRs as confirmed in this research, are major sources of illicit and toxic alcohols. These factors have a major role in the current epidemic of methanol poisoning in Iran. Market surveillance as well as increasing public awareness regarding the methanol-tainted ABHR should be intensified to prevent the ongoing intoxication of the Iranian population by methanol.

## Conclusion

COVID-19 pandemic resulted in development of the Iranian policy for the sale and use of ABHR with little control. As hand hygiene using ABHR is an important preventive measure of disease transmission, in addition to social distancing and vaccines, non-standard ABHR contents may expose the people to risks of COVID-19 infection and methanol poisoning. More control and guidance over the production of ABHR, which is sometimes made in small workshops without the necessary facilities or even in a counterfeit or illegal manner, seems to be necessary. This is particularly the case during infectious outbreaks when the need and consumption of ABHRs is higher, but with less quality control. Those alcohols are not only ineffective, but may also cause methanol toxicity if ingested. Better provision of minimum requirements to companies and ongoing surveillance of contents by the government is warranted.

## Data Availability

The datasets generated and/or analyzed during the current study are available from the. corresponding author on request.
